# Comparison and Mapping Facilitate Relation Discovery and Predication

**DOI:** 10.1371/journal.pone.0063889

**Published:** 2013-06-25

**Authors:** Leonidas A. A. Doumas, John E. Hummel

**Affiliations:** 1 Department of Psychology, University of Hawaii at Manoa, Honolulu, Hawaii, United States of America; 2 Department of Psychology, University of Illinois, Urbana-Champaign, Urbana, Illinois, United States of America; Indiana University, United States of America

## Abstract

Relational concepts play a central role in human perception and cognition, but little is known about how they are acquired. For example, how do we come to understand that physical force is a higher-order multiplicative relation between mass and acceleration, or that two circles are the *same-shape* in the same way that two squares are? A recent model of relational learning, DORA (Discovery of Relations by Analogy; Doumas, Hummel & Sandhofer, 2008), predicts that comparison and analogical mapping play a central role in the discovery and predication of novel higher-order relations. We report two experiments testing and confirming this prediction.

## Introduction

Human thoughts routinely express relations between two or more things. From mundane musings like, “this box won't fit into the trunk of my car” or “my kids won't eat that for dinner”, to more interesting fare such as, “these data are consistent with that hypothesis” or “you can't get a four-way interactions out of a three-variable design”, relational thoughts are ubiquitous in human cognition. Relational concepts underlie our understanding of everything from how to prepare a meal or drive a car to how to solve mathematical, scientific or legal problems. The capacity to understand and reason about abstract relations is a major factor distinguishing adult cognition from the cognitive abilities of children (e.g., [1]–[4]), and, along with language, is the major factor distinguishing human cognition from the cognitive abilities of other animals (including our closest cousins in the animal kingdom; [5]–[7]).

A great deal is known about how people use relational concepts in the service of reasoning, problem solving and everyday life (see, e.g., [5], [8], [9]). By contrast, little is known about where these concepts come from in the first place: How do people acquire the relational concepts that serve as the currency of so much of their cognition? For example, how do we come to understand that *higher-than* is a relation in its own right, so that one object may be higher than another, even if both are low (e.g., close to the floor; [10]), or that two circles are the *same-shape* in the same way that two squares are? This question is important because the vocabulary of relations a person understands has an enormous influence on the kinds of problems that person can and cannot solve. For example, a fundamental difference between an expert physicist and a novice is that the former understands physical relations that the latter does not (e.g., [11]). More fundamentally, understanding how the mind comes to discover relations and represent them as explicit predicates would contribute substantially to our understanding of the origins of human perception and thinking, and to the development of symbolic thought [4].

Acquiring relational concepts is difficult, in part, because (at least in the limit) relations are *invariant* with their arguments: *Smaller-than* means the same thing in *smaller-than* (rhinoceros, elephant) as in *smaller-than* (mouse, rat). It is precisely this invariance that allows relational representations to serve as the basis of analogy (see [5], [6], [9], [12]). However, this invariance makes relational concepts difficult to acquire because all the examples of relations that we encounter in the real world are instantiated with specific arguments: One never gets to observe pure disembodied *smaller-than*-ness; instead, *smaller-than* is always observed as instances of one concrete thing that is smaller than some other concrete thing. Similarly, one does not observe disembodied *loves* or *ameliorates*. Given this, how do people come to represent relations such as *smaller-than*, *loves* or *ameliorates* in a way that is independent of their arguments?

A solution to the problem of how representations of relations can be learned from examples has been proposed in a recent model called DORA (*Discovery of Relations by Analogy*; [13]). The very basic idea behind the DORA model is that when we encounter instances of relations between objects, we compare them to other similar instances we have encountered before. This comparison consists of mapping one instance onto the other. During mapping, any properties common to both instances are aligned, and consequently highlighted. Via intersection discovery the system will learn what the instances have in common (i.e., what is invariant between instances of a relation) while learning to ignore details on which they differ. For example, when DORA observes two instances of chasing – e.g., a dog chasing a cat, and a boy chasing a girl – it will compare and map them. Mapping the dog onto the boy, DORA will allow DORA to, via intersection discovery, abstract any features they share – here the properties of being a *chaser* – and represent those features as an explicit structure that can take arguments (i.e., a predicate). Mapping the cat and the girl will, similarly, allow DORA to learn a representation of *chased*. Although the early representations of relations that DORA learns will be colored by the irrelevant details of the first examples it experiences (e.g., the dog and boy might also share the properties of being male), applied iteratively, intersection discovery will eventually yield representations of relations that are arbitrarily indifferent to their arguments (see [13]). Doumas and colleagues demonstrated that this simple comparison-based learning algorithm accounts for more than 35 major findings in the literatures on cognitive development and relational learning in both children and adults (e.g., [13]–[19]).

One of DORA's key theoretical insights is that comparison plays a central role in the discovery of novel relational concepts. That is, to learn a novel relation, two instances of the relation must be compared, and corresponding elements of the two relations (i.e., those elements playing the same roles) must be mapped. For example, to learn a relation like *pushes*, two instances of pushing must be compared such that the two elements doing the *pushing* are mapped and the two elements being *pushed* are mapped. DORA thus predicts that a relation cannot be learned from a single instance. However, it is important to note here that this prediction does not imply that DORA must observe or experience any specific instance of a relation to learn that relation or represent that instance. DORA learns representations from specific instances and refines these representations over experience with future instances. However, once DORA has learned a relation it can apply that relation to any applicable instances in the future. For example, DORA learns a relation like *chase* (*x*, *y*), it can apply that relation to future instance of chasing, including instances involving objects it has never seen chasing one-another (e.g., representing that *chases* (teapot, teacup)), or even objects it has no experience with (e.g., representing that *chases* (galoop, grindel), where a galoop and a grindel are novel objects). More formally, DORA can learn a relation R(A,B), with {(a,b) a element of A, b element of B} with experience of only a small set of specific a,b pairings. See [13] for several demonstrations of this point.

Consistent with this hypothesis, several studies have demonstrated that making analogical mapping (i.e., a structured comparison) bootstraps the induction of relational schemas (e.g., [3], [20]–[22]), and that comparison, a cousin of mapping, can help people discover which features and relations are relevant in a given task ([23]–[30]). While many specific theories of mapping exist (e.g., [5], [9], [12], [13], [31]), they all fall generally under the umbrella of [32] structure mapping theory. According to structure mapping theory when an analogy is made between two domains (a target and a source) similar predicates representing object properties and relations are aligned and explicit connections or mappings are made between them. Arguments of mapped predicates are then themselves aligned and mapped. For example, when making an analogy between a situation where a dog chases a cat – represented as *chase* (dog, cat) – and another where a boy chases a girl – represented as *chase* (boy, girl) – the two *chase* predicates will be aligned and mapped. Based on the mapping between the two *chase* predicates dog and boy will be aligned and mapped (by virtue of both being agents of the *chase* relation) and the cat and girl will be aligned and mapped (by virtue of both being patients of the *chase* relation). While various mapping theories put different amounts of weight on how factors such as systematicity (preferring mappings between systems of relations) and pragmatics will influence the alignments and subsequent mappings, the general format of aligning predicates followed by aligning their arguments central to structure mapping theory is accepted widely.

The current experiments were designed to investigate DORA's prediction that comparison bootstraps the discovery of novel relations. Because finding simple relations that are unknown to adult humans is difficult, the current studies focused on the discovery of novel higher-order relations (i.e., relations whose arguments are themselves relations).

## Overview of the Experiments

We present two experiments investigating DORA's predictions about the role of comparison in the discovery and predication of novel higher-order relations. Both experiments used category learning as an index of relational learning: Categories were defined by novel higher-order relations among an exemplar's elements (as elaborated shortly). The categories were constructed so that only by discovering the relevant higher-order relation could participants achieve above-chance categorization performance. Accordingly, participants' categorization performance served as an index of learning the higher-order relation.

Experiment 1 was conducted to test of the role of comparison in participants' ability to discover novel relations. The stimuli were designed so that the relevant first-order relations (those over which the category defining novel higher-order relations were defined) would be salient to undergraduates (our participant population). Experiment 2 acted as a more stringent test of the role of comparison, per se, in the learning and predication of novel relations. It used the same basic paradigm as Experiment 1, but with more abstract stimuli and an additional manipulation. Specifically, the stimuli were designed to make the the comparison task difficult, so that some participants would fail to find the correct correspondences and thus fail to discover the category-relevant higher-order relation.

### Ethics Statement

Human subjects approval was obtained for both experiments from the University of California, Los Angeles (UCLA) Institutional Review Board operating from the UCLA Office of the Human Research Protection Program. All participants provided written consent before participation.

## Experiment 1

Experiment 1 served as a basic test of DORA's prediction about the role of comparison in participants' ability to discover novel relations. As noted above, DORA predicts that in order to discover a novel relation it should be necessary and sufficient to compare two situations and map the corresponding elements over which the novel relation holds. That is, mapping two exemplars in which a novel relation holds should lead to the discovery and predication of that relation. However, in the absence of mapping, the relation should remain undiscovered even when participants are otherwise exposed to it.

**Figure 1:**
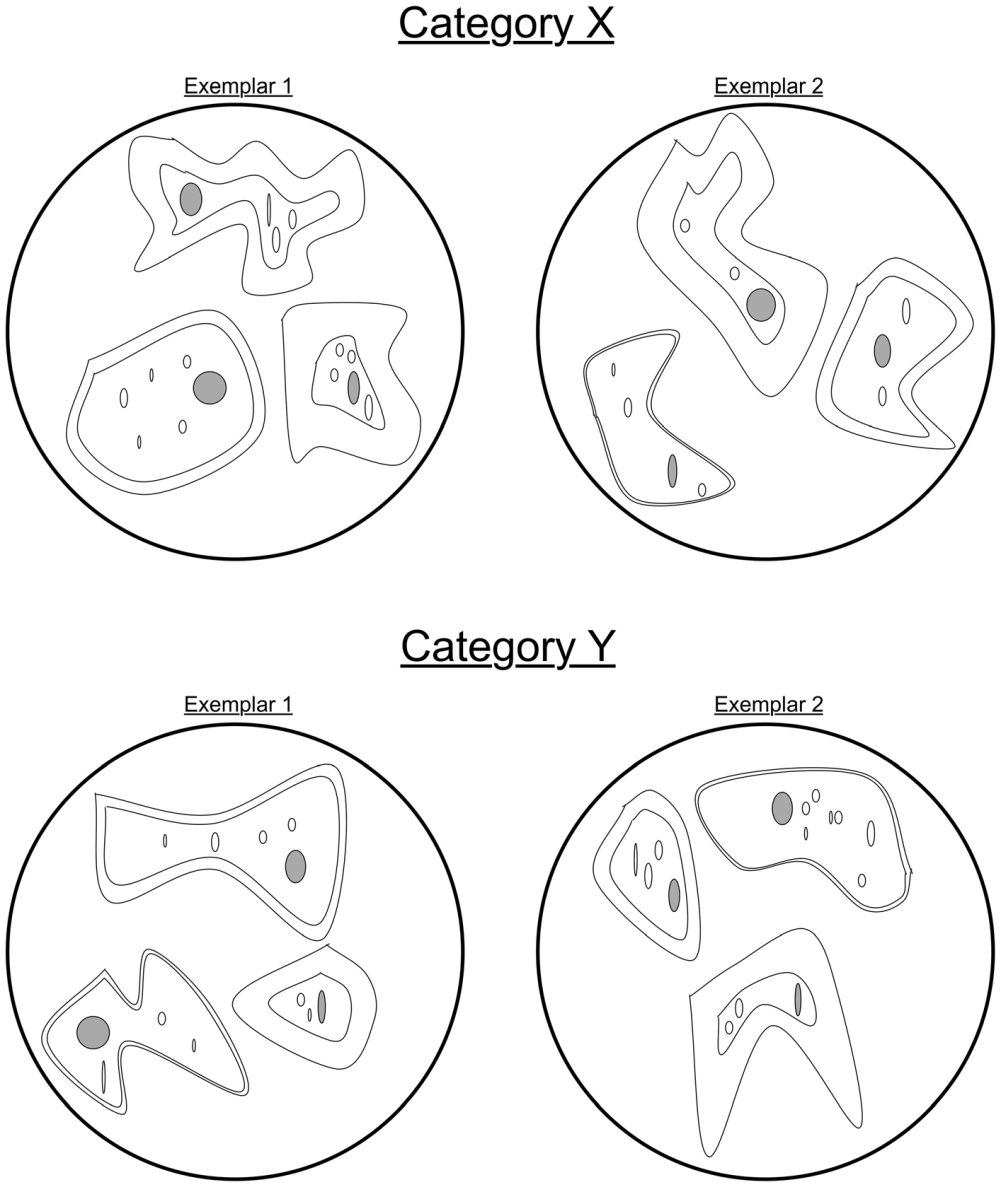
Examples of the stimuli used in Experiment 1. The top row contains two exemplars from Category X; the bottom row contains two exemplars from Category Y. Each exemplar consists of three cells in a circle. Cells differ in their size, their location in the circle, the thickness of their membranes (the outer wall of the cell), the roundness of their nuclei (the large grey oval in each cell), the number of organelles in the cell (the small white ovals). In exemplars from Category X, cells with thicker membranes also have rounder nuclei. In exemplars from Category Y, cells with thicker membranes also have more elongated nuclei.

In order to test for relation discovery, we defined novel (to the participants) higher-order relations and used these relations to define two categories. Exemplars consisted of drawings of three simple “cells” inside a circular frame (see Figure 1). Within an exemplar, the cells varied in their location in the frame, their shape, the thickness of their membrane, the roundness of their nucleus (large grey oval), and the number of organelles (smaller white ovals). Categories were defined by a higher-order relation between the cells' membrane thickness and the roundness of their nuclei: In Category X, the thicker a cell's membrane, the rounder its nucleus; in Category Y, the thicker the membrane the more elliptical its nucleus. The cells' locations in the frame, shape, and number of organelles varied randomly and were uncorrelated with category membership.

**Figure 2:**
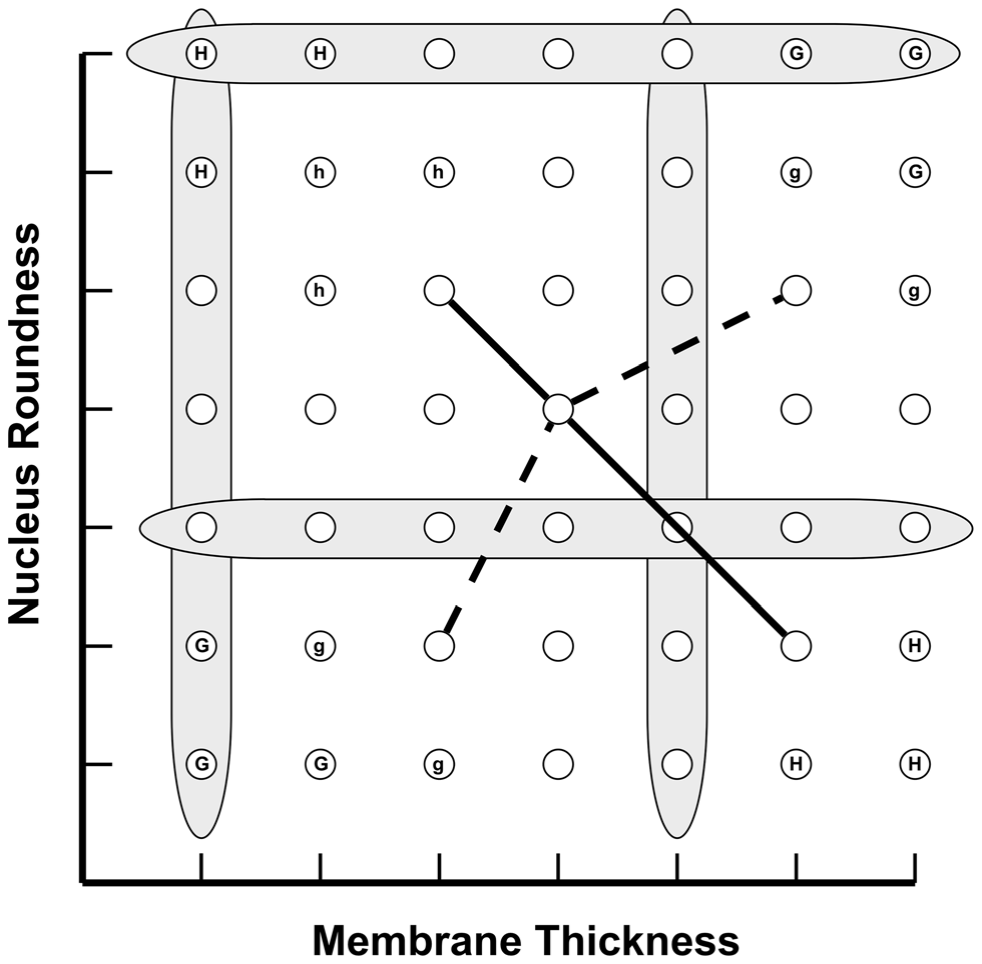
Analysis of the category structure of Experiment 1. Rows correspond to values of “nucleus roundness”; columns correspond to values of “membrane thickness.” Any cell resides at one entry in the matrix (circles in the matrix). A single exemplar consists of three cells (i.e., three circles from the matrix). Members of X consist of three cells connected by lines of finite positive slope (such as the dashed line). Members of Y consist of three cells connected by lines of finite negative slope (such as the solid line). Gray bars indicate values withheld from training exemplars for use during transfer. Circles marked “G” correspond to cells that can appear only in category X; circles marked “g” appear only in X during training, but in either X or Y during transfer. Cells marked “H” only appear in category Y; those marked “h” appear only in Y during training, but in either X or Y during transfer. Values “g” and “h” are misleading in that a participant who learns to categorize based on them (i.e., based on feature conjunctions) will make systematic errors during transfer. Unmarked cells appear with equal likelihood in X or Y.

The exemplars were designed to make category learning impossible without discovering the higher-order relation between relative membrane thickness and nucleus roundness. To this end, absolute thickness and roundness were non-predictive of category membership, as were relative thickness and relative roundness in isolation (i.e., in every exemplar of both categories, some membranes were thicker than others and some nuclei were rounder than others; see Figure 2). It is only possible to achieve ceiling-level categorization performance using the higher-order relation between relative thickness and relative roundness to define category members: If, as the cells' membranes got thicker (relative to the other cells in the exemplar) their nuclei got rounder (relative to the other cells), then the exemplar belonged to X; if the opposite held, it belonged to Y. These category-defining higher-order relations were chosen because they were unlikely to be familiar to undergraduates prior to the experiment (and hence learnable during the course of the experiment), although the relevant first-order relations are likely to be salient. In addition, as elaborated below, we also conducted extensive pretesting with the categories to make sure that participants did not already know the category-defining relations.

Our hypothesis was that comparing two stimuli from the same category and mapping corresponding elements from the stimuli onto one another (as elaborated below) would cause participants to discover and predicate the critical higher-order relation defining their category membership. If the hypothesis is correct, then categorization performance should improve markedly after performing such a comparison (relative to performance prior to comparison). To the extent that mapping facilitates discovery and predication of the category-defining higher-order relations, post-mapping categorization performance in the Map condition should go rapidly to ceiling, whereas post-study categorization performance in NoMap should remain near chance.

### Methods

#### Participants

20 undergraduates (10 in Map; 10 in NoMap) participated for course credit.

#### Materials

Seven membrane thicknesses and seven nucleus roundnesses were used to construct the stimuli. As a result, the thickest membrane (or roundest nucleus) in one exemplar of a category could be the thinnest (or most elliptical) in another exemplar of the same category. It was therefore impossible to categorize correctly based on absolute membrane thickness and nucleus roundness (see Figure 2).

Membrane thicknesses 3 and 7 (the thickest) and nucleus roundnesses 1 (the least round) and 5 were excluded from the exemplars presented during the pre-mapping, post-mapping, and mapping phases, reserving them for use on the transfer trials (elaborated directly below). The transfer exemplars were created as described above, with the additional constraints that at least one novel thicknesses and one novel roundness appeared in each exemplar, and each novel thickness and roundness appeared in at least three of the six transfer exemplars. The withheld thicknesses and roundnesses consisted of values both within and outside the bounds of the values seen during the training and test phases, and thus required participants to both interpolate and extrapolate to new values.

#### Procedure

Participants received 40 pre-mapping training trials (20 Xs and 20 Ys) in a random order. Their task was to indicate whether each belonged to Category A or B (the labeling of X and Y as A or B was counterbalanced). They received feedback on every trial indicating whether they had categorized the exemplar correctly or incorrectly. To the extent that the category-defining relations are unfamiliar, performance should be near chance during these trials. Following these trials, half the participants – those in the *Map* condition – performed a comparison task, and the other half of the participants – those in the *NoMap* condition – performed a study task. Participants in Map condition were shown two exemplars of the same category (X or Y, counterbalanced) and asked to compare the two instances and indicate which cell in one exemplar corresponded to which in the other and to explain why (i.e., to map the elements in one exemplar to elements in the other). Participants were informed that the exemplars came from the same category but they were not told which. A participant was considered to have mapped correctly if during the mapping task she aligned corresponding cells from the exemplars. That is, if both exemplars where from Category X, then a correct mapping would be to align the two cells with the thickest membranes and roundest nuclei one onto the other (i.e., to align the cell from the first exemplar with the thickest membrane and roundest nucleus to the cell in the second exemplar with the thickest membrane and the roundest nucleus), the cells with the thinnest membrane and most elongated nuclei one onto the other, and the cells with the middlemost membrane thickness and nucleus roundness one onto the other. Alternately, if both exemplars where from Category Y, then a correct mapping would be to align the two cells with the thickest membranes and most elongated nuclei one onto the other, the cells with the thinnest membrane and most round nuclei one onto the other, and the remaining two cells one onto the other. Participants were given no feedback about the correctness of their mapping. Participants in the NoMap condition viewed the same exemplars, but were not instructed to map them onto one another; instead, they were told that the exemplars came from the same category and instructed to study them for one minute. All participants then received 40 post-mapping training trials with feedback. In the transfer phase participants viewed six transfer exemplars (3 Xs and 3 Ys) in a random order. Their task was to categorize each without feedback. After the transfer trials participants were asked what rule (if any) they had used to categorize the exemplars.

## Results and Discussion

The results were as predicted. For the pre-mapping trials, an independent-samples t-test revealed no effect of mapping, t(18) = 1.13, p>25, which is expected, as the groups received identical treatment prior to mapping. Moreover, neither group's performance differed reliably from chance.

**Figure 3:**
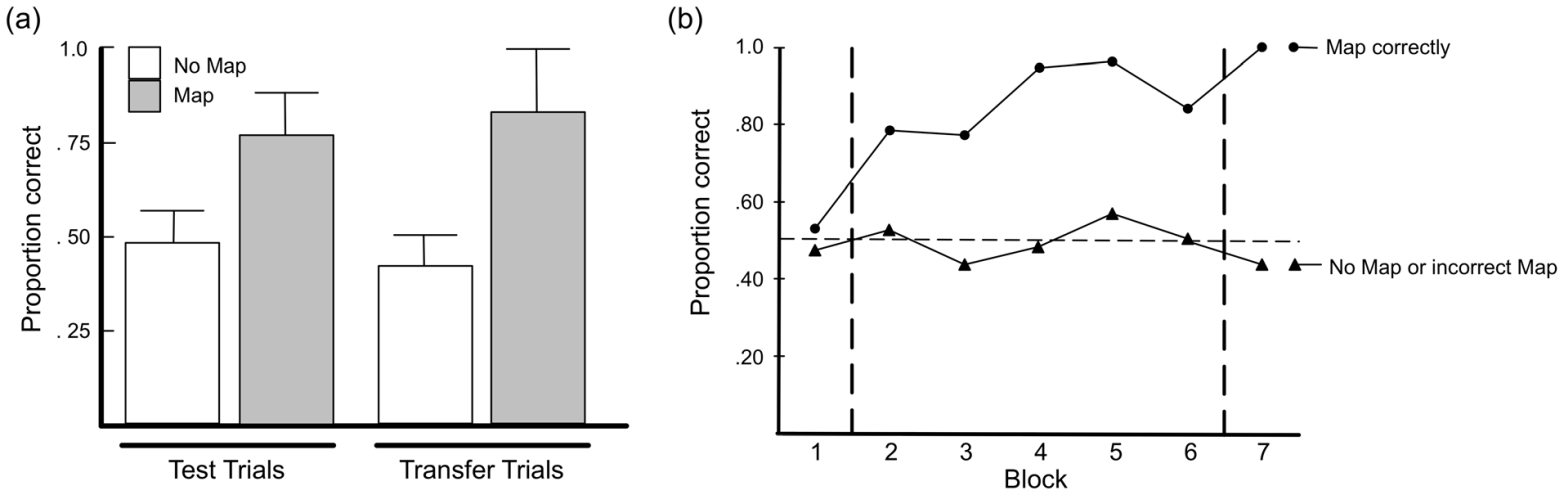
Probability of correct response on post-mapping and transfer trials as a function of condition in Experiment 1 (a). The dashed line indicates chance. (b) Probability of correct response on pre-mapping trials (PM), successive blocks of post-mapping trials (8 trials per block; “1…5”), and transfer trials (T) as a function of mapping performance in Experiment 1.

An independent-samples t-test on the post-mapping trials (Figure 3a) revealed that accuracy in the Map condition (mean  = 77) was reliably higher than in NoMap (mean  = 48), t(18) = 3.84, p<01. Accuracy in NoMap did not differ from chance.

A similar pattern obtained on the transfer trials (Figure 3a). Accuracy in Map was reliably greater (mean  = .83) than in NoMap (mean  = 42), t(18) = 4.16, p<01. Performance in NoMap did not differ from chance.

Figure 3b depicts the trial-by-trial data for participants who mapped correctly (7 participants from Map) against those who did not (3 from Map and 10 from NoMap). Initially all participants were at chance. However, after the mapping phase the performance of participants who mapped correctly climbed rapidly to ceiling, whereas participants who did not map correctly remained at chance throughout the experiment.

Participants' reports of their mappings and rule use also revealed an interesting pattern. None of the participants in NoMap, and 7 participants in Map correctly stated the rule defining category membership at the end of the experiment. All and only those participants who correctly mapped the elements during the mapping phase correctly stated the category-defining relation. All other participants either missed the relevant dimensions completely or categorized based on absolute membrane thickness and absolute nucleus roundness, which, as stated previously, was not sufficient for correct categorization.

In Experiment 1, all and only those participants who correctly mapped exemplars to one another went on to correctly categorize them during the post-mapping categorization and transfer phases. Experiment 2 was designed as a more stringent test of the necessity and sufficiency of mapping for relational discovery and predication.

## Experiment 2

**Figure 4:**
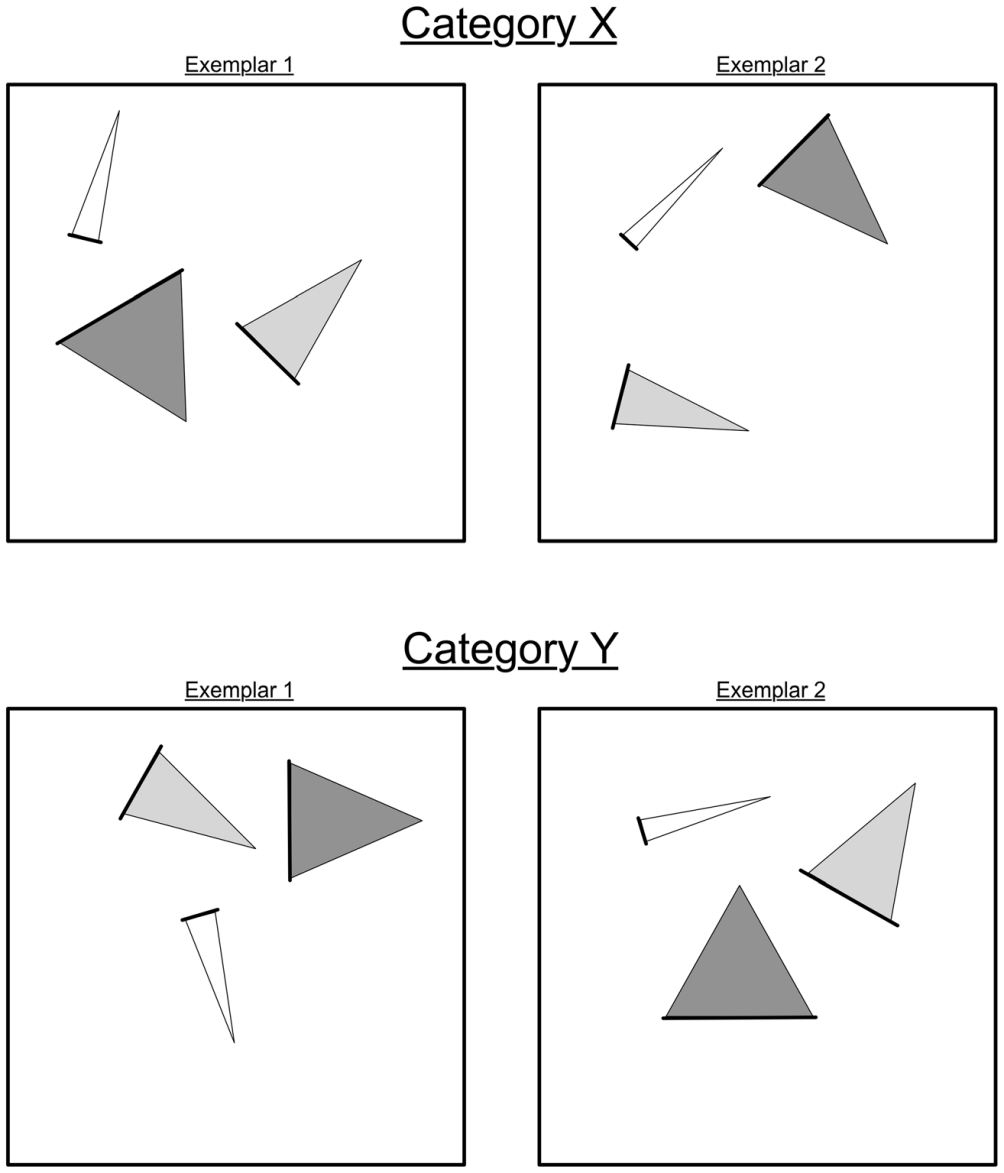
Examples of the stimuli used in Experiment 2. The top row contains two exemplars from Category X; the bottom row contains two exemplars from Category Y. Each exemplar consists of three triangles in a square-frame. Triangles differ in their location in the square-frame, their color, the width of their base (marked by the bold line), and their orientation from 0-degrees. In exemplars from Category X, triangles with thicker bases are more misoriented from 0-degrees. In exemplars from Category Y, triangles with thicker bases are less misoriented from 0-degrees.

Experiment 2 served as a more stringent test of the role of mapping, per se (as opposed to other cognitive processes that may go on during mapping), in the learning and predication of novel relations. It used the same basic paradigm as Experiment 1, but with different stimuli and an additional manipulation. Each exemplar in Experiment 2 consisted of three isosceles triangles inside a square frame (see Figure 4). The triangles differed in their location inside the frame, their color (one was red, one blue and one green) their width at their base and their orientation. The category-defining relation was the higher-order relation between the triangles' relative orientations and relative width: In category X, the more a triangle was rotated away from the upright, the wider it was at its base, whereas in category Y, the closer a triangle was to upright, the wider it was at its base. The triangles' locations inside the frame were non-diagnostic and their colors were semi-diagnostic. These stimuli were designed to make the mapping difficult, so that most participants would fail to find the correct mapping by default. In order to use a relation as a basis for mapping, it is necessary to predicate that relation (i.e., to represent it as an explicit structure that can take arguments; see [5], [9], [12], [13], [31]). However, pilot work revealed that undergraduates did not, by default, predicate a triangle's orientation in the picture plane.

In this experiment, prior to the Map or (NoMap) study task, half the participants (the *Difference-identification* (*DI*) group) were given a task in which they were asked to state how the triangles within a single exemplar differed from one another. This task was designed to cause the participants to predicate the relevant first-order relations in the exemplars (as elaborated below). The other half of the participants (the *No-difference-identification* (*ND*) group) were simply instructed to study an exemplar for one minute. Following the DI or (ND) study task, participants participated in the Map/NoMap tasks as in Experiment 1. DI vs. ND was crossed orthogonally with Map vs NoMap.

The reasons for the additional manipulations were two-fold. First, to the extent that (a) explicit predication of the relevant first-order relations is necessary for successful mapping, and (b) successful mapping is necessary for relation discovery, only those participants in both the Compare and Map conditions should discover the critical higher-order relation, and only they should be able to categorize the stimuli during post-mapping categorization and transfer.

Second, one potential criticism of experiment 1 was that it was not mapping, per-se, that led participants to discover the relevant relations, but rather some other cognitive process associated with mapping, such as talking about the exemplars, or comparing items within an exemplar rather than between exemplars. By introducing the second DI/ND condition we controlled for these extraneous cognitive operations. If indeed simply talking about exemplars or comparing items from within a single exemplar is sufficient to lead participants to predicate the novel relations, then participants in the DI group should predicate the relevant relations whether or not they perform the mapping task. On the other hand, if mapping is a necessary component of predication of novel relations, then just as in Experiment 1, only participants who perform the mapping task correctly should reach above-chance categorization performance.

### Methods

#### Participants

64 undergraduates (16 per condition) participated for course credit.

#### Materials

Each exemplar consisted of three isosceles triangles (Figure 4), which differed in width, orientation, color and location. To make the triangles' orientations unambiguous, the base of each triangle was marked with a bold line. There were 13 orientations (from upright, 0°, to 180° in 15° increments) and 13 widths. To make the triangles discriminable, no two triangles in any exemplar were of adjacent widths or orientations. These constraints made it possible for the most misoriented/widest triangle in one exemplar of a category to be the most upright/narrowest in another, making it impossible to categorize correctly based on the triangles' absolute orientations or widths.

The locations of the triangles in an exemplar varied randomly (participant to the constraint that they not overlap), and their colors covaried imperfectly with category membership: In 80% of Category X, the widest triangle was red and the narrowest blue; in 20% this relationship was reversed. In category Y this relationship was reversed. In all exemplars, the middle-width triangle was green.

**Figure 5:**
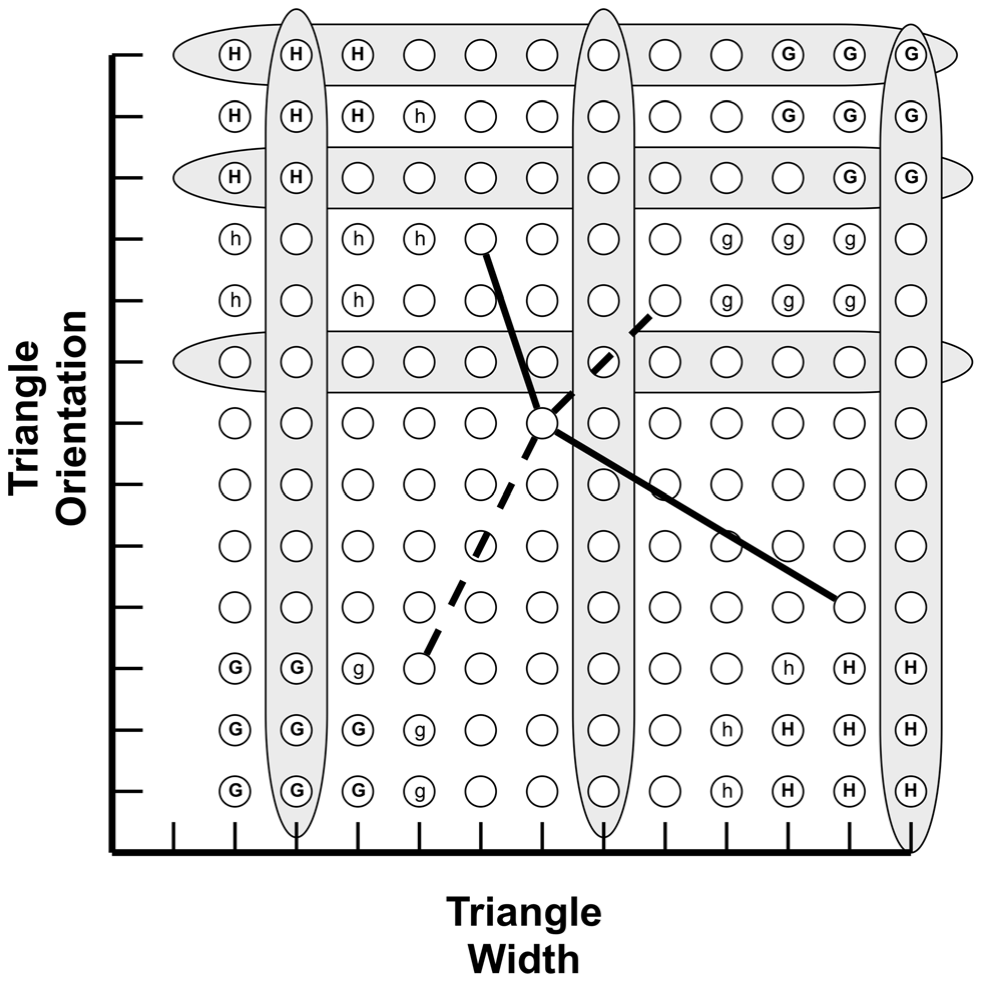
Analysis of the category structure in Experiment 2. The logic is identical to that of the matrix in Figure 2.

We withheld widths 2, 7 and 13, and orientations 8, 11 and 13 for construction of transfer exemplars. Transfer exemplars were constructed as described above, with the additional constraint that at least two novel widths and two novel orientations appeared in each. The withheld widths and orientations consisted of values both within and outside the bounds of the values seen during training, requiring participants to both interpolate and extrapolate during transfer (see Figure 5).

#### Design and Procedure

DI vs. ND and Map vs. NoMap (see above) were crossed orthogonally, resulting in four between-participants conditions. All participants first received 40 pre-mapping categorization trials in random order. Following these trials, participants in the DI condition were shown two exemplars of the same category and instructed to state how each triangle in the exemplar on the left differed from the other triangles in that exemplar. Those in the ND condition were instructed to study the exemplar for one minute. Next, the Map and NoMap conditions proceeded as in Experiment 1. All participants then performed 20 post-mapping categorization trials with feedback, followed by six transfer trials without feedback.

## Results and Discussion

The results were again as predicted. For the pre-mapping trials, a two-way between participants ANOVA showed no main effects of either Map/NoMap, F(1, 60) = .023, p>05, or DI/ND, F(1, 60) = 211, p>05. There was a slightly reliable mapping-by-comparison interaction, F(1, 60) = 5.273, p<05, but Bonferroni post-hoc tests (α = 5) revealed no significant differences between any groups. No group differed reliably from chance in their pre-mapping categorization performance.

**Figure 6:**
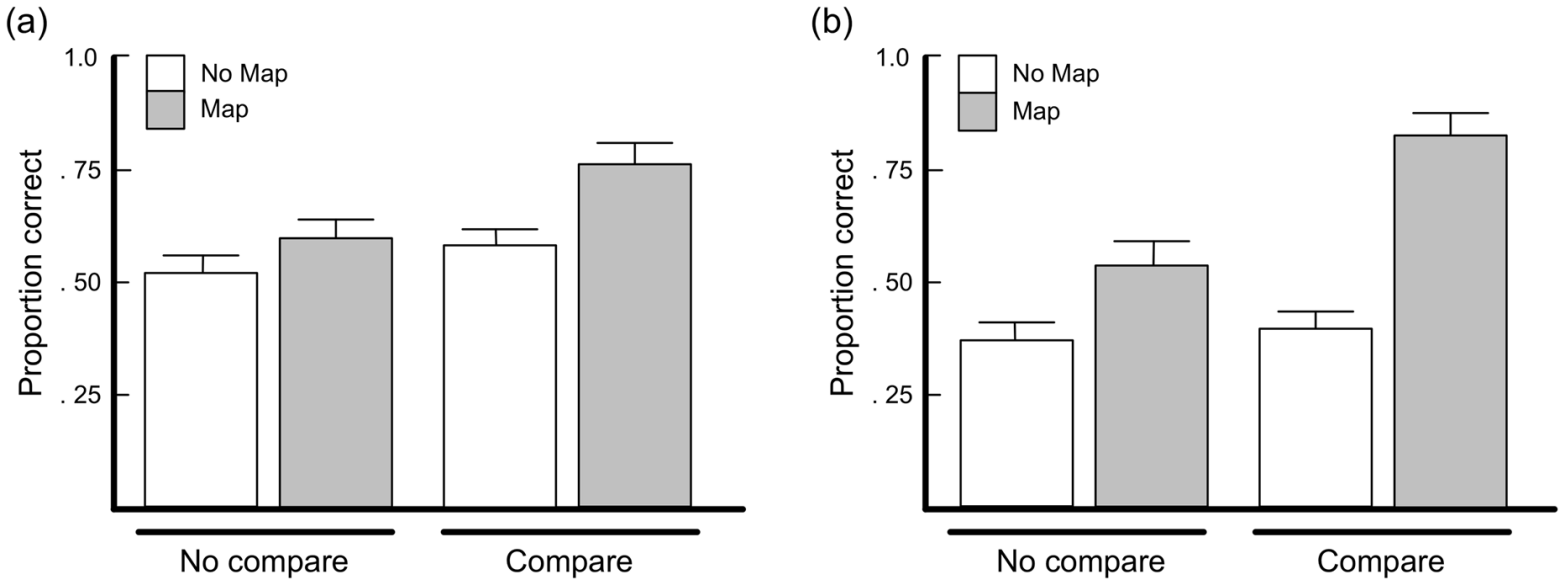
Probability of correct response on post-mapping trials as a function of condition in Experiment 2 (a). (b) Probability of correct response on transfer trials as a function of condition in Experiment 2.

A different pattern appeared after the mapping condition, however (see Figure 6a). A two-way ANOVA on post-mapping categorization trials revealed that participants who mapped categorized reliably more accurately than those than those who did not, (68% vs. 55%) F(1, 60) = 11.607, p<01. A similar pattern obtained for participants in DI vs. ND, (67% vs. 56%) F(1, 60) = 8.978, p<01. There was no reliable interaction, F(1,60) = 2.064, p>05. Post-mapping classification accuracy was higher in DI/Map than in the other three conditions. A Bonferroni post-hoc test at the α = 05 level revealed a reliable difference between DI/Map and both ND/NoMap (ΔM = 4.88, t(30) = 4.369, p<01) and DI/NoMap (ΔM = 3.69, t(30) = 3.304, p<01). The difference between DI/Map and ND/NoMap approached reliability (ΔM = 3.38, t(30) = 2.694, p = 011), but did not meet the set Bonferroni criterion. No differences between the other three groups were reliable. Post-mapping classification accuracy was no greater than chance in any condition except Compare/Map.

A similar pattern obtained on the transfer trials (Figure 6b). The effects of Map/NoMap, F(1, 60) = 35.799, p<01, and DI/ND, F(1, 60) = 10.274 p<01, and the Map/NoMap-by-DI/ND interaction, F(1, 60) = 7.717, p<01, were all reliable. A Bonferroni post-hoc analysis revealed a reliable difference between DI/Map and ND/NoMap (ΔM = 2.75, SE = .45, p<01), ND/Map (ΔM = 2.00, SE = 45, p<01), and DI/NoMap (ΔM = 2.00, SE = 45, p<01), and no differences between any other groups. Again only performance in the DI/Map condition was greater than chance.

The participants' reports of their rule use revealed the same pattern as observed in Experiment 1. None of the 32 participants in ND/NoMap and DI/NoMap, 3 participants in ND/Map and 14 participants in WI/Map correctly stated the rule defining category membership at the end of the experiment. As in experiment 1, there was a 1∶1 correspondence between participants who performed the mapping task correctly and those who correctly stated the rule (every participant who mapped correctly also correctly stated the rule at the end of the experiment). These results are predicted by the hypothesis that mapping, per se, facilitates the discovery and predication of novel higher-order relations.

## Discussion

Relations play a central role in human perception and thinking, yet little is known about how relational concepts are acquired. The results of two experiments suggest that comparison and mapping facilitate the discovery and predication of novel higher-order relations. In both experiments, participants who successfully mapped exemplars from the same category onto one another learned a novel, category-defining higher-order relation between their elements and no participant who failed to map correctly succeeded in learning the relation. Indeed, categorization performance of the latter group never got above chance.

Importantly, participants who successfully mapped were able to both interpolate and extrapolate learning to new exemplars with novel stimulus values (i.e., novel membrane thicknesses and nucleus roundnesses in Experiment 1 and novel orientations and widths in Experiment 2) and to verbally state the relational rules defining category membership. Participants who did not map were unable to either transfer to new stimuli or to state the rule. In line with DORA's prediction, these findings suggest that mapping bootstraps the discovery of novel relations, and that the resulting relations are represented explicitly, in the sense of being available to bind to novel inputs (see [9], [12], [13], [33]).

In addition, the results of Experiment 2 support to the prediction made by all structure-based models of analogy that explicit predication of the relevant relations is necessary for successful structure mapping (e.g., [5], [9], [12], [13], [31]). More specifically, we found that discovering that a higher-order relation applied in a particular instance required mapping the component lower-order relations that act as arguments of that higher-order relation (e.g., discovering that the higher-order *covaries* relation applies to the relations *wider-than* and *more-misoriented* about triangles requires mapping based on those lower-order relations). Successfully mapping instances based on lower-order relations (e.g., mapping two triangles that are both *most-misoriented* and *most-wide*) requires first representing those lower-order relations.

The findings reported here suggest that the same cognitive mechanisms that underlie our ability to make analogies – namely, those underlying structure mapping – also underlie our ability to discover and predicate the relational concepts that support those mappings. If this suggestion is correct, then the evolution of the capacity for generalized structure mapping may well be the “great leap forward” [34] that ultimately gave rise to our capacity for generalized symbolic thought [6].
